# Pathogenesis of Non-Infectious Uveitis Elucidated by Recent Genetic Findings

**DOI:** 10.3389/fimmu.2021.640473

**Published:** 2021-04-12

**Authors:** Masaki Takeuchi, Nobuhisa Mizuki, Shigeaki Ohno

**Affiliations:** ^1^ Department of Ophthalmology and Visual Science, Yokohama City University Graduate School of Medicine, Yokohama, Japan; ^2^ Department of Ophthalmology, Faculty of Medicine and Graduate School of Medicine, Hokkaido University, Sapporo, Japan

**Keywords:** acute anterior uveitis, Behcet’s disease, birdshot chorioretinopathy, GWAS - genome-wide association study, immunogenetics, ocular sarcoidosis, uveitis, Vogt-Koyanagi-Harada disease

## Abstract

Uveitis is a generic term for inflammation of the uvea, which includes the iris, ciliary body, and choroid. Prevalence of underlying non-infectious uveitis varies by race and region and is a major cause of legal blindness in developed countries. Although the etiology remains unclear, the involvement of both genetic and environmental factors is considered important for the onset of many forms of non-infectious uveitis. Major histocompatibility complex (MHC) genes, which play a major role in human immune response, have been reported to be strongly associated as genetic risk factors in several forms of non-infectious uveitis. Behçet’s disease, acute anterior uveitis (AAU), and chorioretinopathy are strongly correlated with MHC class I-specific alleles. Moreover, sarcoidosis and Vogt-Koyanagi-Harada (VKH) disease are associated with MHC class II-specific alleles. These correlations can help immunogenetically classify the immune pathway involved in each form of non-infectious uveitis. Genetic studies, including recent genome-wide association studies, have identified several susceptibility genes apart from those in the MHC region. These genetic findings help define the common or specific pathogenesis of ocular inflammatory diseases by comparing the susceptibility genes of each form of non-infectious uveitis. Interestingly, genome-wide association of the interleukin (IL)23R region has been identified in many of the major forms of non-infectious uveitis, such as Behçet’s disease, ocular sarcoidosis, VKH disease, and AAU. The interleukin-23 (IL-23) receptor, encoded by *IL23R*, is expressed on the cell surface of Th17 cells. IL-23 is involved in the homeostasis of Th17 cells and the production of IL-17, which is an inflammatory cytokine, indicating that a Th17 immune response is a common key in the pathogenesis of non-infectious uveitis. Based on the findings from the immunogenetics of non-infectious uveitis, a personalized treatment approach based on the patient’s genetic make-up is expected.

## Introduction

Uvea is the middle layer of three concentric layers of the eye, including the iris, ciliary body, and choroid. Uvea plays an important role in providing diffusible nutrients in the outer layer of the retina and homeostasis of the temperature of the eye. Conversely, because of the abundant blood flow, it tends to be the primary site of inflammatory reaction in the eye, leading to uveitis. Uveitis can be clinically divided into infectious uveitis and non-infectious uveitis. Non-infectious uveitis often develops as an ocular symptom of systemic disease.

Uveitis is one of the major causes of visual impairment in developed countries. Severe eye inflammation irreversibly damages the retina and optic nerve. Uveitis accounts for 5%–20% of legal blindness in the United States and Europe and leads to irreversible blindness in approximately 35% of patients in developed countries (1, 2).

Although the pathophysiology of non-infectious uveitis has not yet been clarified, the involvement of genetic predisposition and environmental factors has been suggested. With the recent development of genome analysis research, several susceptibility genes involved in the pathophysiology of each non-infectious uveitis have been identified, contributing to the understanding of the pathophysiology of non-infectious uveitis. Here we describe immunogenetics of non-infectious uveitis elucidated by genetic analysis focusing on genome-wide association studies (GWASs).

## Genetic Evidence of Non-Infectious Uveitis

### Behçet’s Disease

Behçet’s disease (BD) is a systemic inflammatory disease with repeated attacks and remissions that causes inflammation of various organs of the body, including oral aphtous ulcers, uveitis, skin lesions, and genital ulcers (3). BD is relatively common in countries that fall on the ancient silk route, including the Mediterranean basin, Middle East, Central Asia, and East Asia. BD is genetically one of the most studied causes of non-infectious uveitis. The geoepidemiological features of BD suggest that both environmental and genetic factors are likely involved in the development of BD. Familial aggregation and high sibling recurrence observed in multiple populations also support the involvement of genetic factors in its pathogenesis (4, 5).

In 1973, Ohno et al. reported a strong association of HL-A5 (later termed HLA-B5) in the Japanese population. HLA-B5 is a broad antigen subsuming HLA-B*51 and HLA-B*52 (6). After finding this initial genetic evidence, the association between BD and HLA-B*51 has been confirmed in multiple populations (7–18). A systematic review and meta-analysis including 4,800 patients and 16,289 controls from 78 independent studies reported the pooled odds ratio for the susceptibility of HLA-B5/HLA-B51 and HLA-B*51, 5.78 (95% confidence interval [CI], 5.00–6.67) and 5.90 (95% CI, 4.87–7.16), respectively. Other than HLA-B*51, other MHC class I alleles also show some disease susceptibility (19). A GWAS targeting microsatellites by Meguro et al. identified HLA-A*26 susceptibility in the Japanese population (20). Due to the strong linkage disequilibrium, it is difficult to reveal the true association of HLA alleles. Stepwise conditional analysis of Turkish GWAS-predicted HLA alleles revealed associations of HLA-B*15, HLA-B*27 as risk alleles and HLA-A*03 as protective alleles, which were independent from HLA-B*51 susceptibility (20, 21).

The summary of susceptible single nucleotide polymorphisms (SNPs) outside of the MHC region for diseases with non-infectious uveitis with P<5 × 10^−8^, thus exceeding the threshold for genome-wide significance, is shown in **Table 1**. In 2010, Mizuki et al. and Remmers et al. conducted GWASs for BD in the Japanese and Turkish populations, respectively. The GWASs initially reported genome-wide associations in loci of *IL23R-IL12RB2* and *IL10* by GWASs in the Japanese and Turkish populations, respectively (22, 23). Genetic association of *UBAC2* was reported in a candidate gene approach based on results of the previous GWAS by a meta-analysis of Turkish and Italian populations, although the p-value did not achieve a general threshold of genome-wide association (P=1.69 × 10^-7^) (48). The susceptibility of *UBAC2* were confirmed in Han Chinese and Japanese populations (49, 50).

**Table 1:** Summary of susceptibility SNPs with genome-wide significance for non-infectious uveitis.

Traits	Variant	Gene	OR	Population		Function of the risk allele	Ref
				Discovery	Replication		
BD	rs1495965	*IL23R-IL12RB2*	1.35	Japanese	Turkish		(22, 23)
BD	rs924080	*IL23R-IL12RB2*	1.28	Turkish, Japanese			(22, 23)
BD	rs10889664	*IL23R*	2.00	Spanish			(24)
BD	rs1518111	*IL10*	1.45	Turkish	Greek, UK, Iranian, Middle Eastern Arab, Japanese, Han Chinese	Reduces expression in monocytes	(23)
BD	rs1800871	*IL10*	1.45	Japanese	Turkish, Korean, Han Chinese		(22, 25)
BD	rs3783550	*IL1A-IL1B*	1.33	Turkish		rs4420765 (r2 = 0.97) increases IL-1β and decreases IL-1α in PBMCs	(26)
BD	rs7574070	*STAT4*	1.27	Turkish	Japanese, Iranian	Increases expression	(27, 28)
BD	rs897200	*STAT4*	1.45	Han Chinese		Increases expression of *STAT4* and *IL17*	(29)
BD	rs17006292	*TFCP2L1*	4.17	Han Chinese			(29)
BD	rs7616215	*CCR1*	1.39	Turkish	Japanese, Iranian	Decreases expression in monocytes Reduces monocyte chemotaxis	(27, 28)
BD	rs13092160	*CCR1-CCR3*	3.13	Han Chinese		Decreases expression in PBMCs	(30)
BD	rs17810546	*IL12A*	1.66	Turkish, Mixed populations			(31)
BD	rs1874886	*IL12A*	1.61	Spanish			(24)
BD	rs17482078	*ERAP1^1^*	4.56	Turkish	Iranian	Reduces enzymatic activity	(27, 28)
BD	rs9494885	*TNFAIP3*	1.81	Han Chinese		No difference in expression in PBMCs	(32)
BD	rs2230801	*RIPK2*	1.53	Turkish, Iranian, Japanese		Possibly damaging (I259T)	(26)
BD	rs10094579	*RIPK2*	1.32	Turkish, Han Chinese			(26, 33)
BD	rs224127	*ADO-ZNF365-EGR2*	1.27	Turkish, Japanese	Han Chinese		(26, 33)
BD	rs1509966	*ADO-ZNF365-EGR2*	1.13	Turkish, Iranian			(26)
BD	rs2848479	*JRKL-CNTN5*	1.66	Spanish			(24)
BD	rs2617170	*KLRC4*	1.28	Turkish	Japanese, Iranian		(27, 34)
BD	rs2121033	*LACC1*	1.32	Turkish, Iranian, Japanese		r2 = 0.93 with a missense variant (I254V)	(26)
BD	rs9316059	*LACC1*	1.37	Japanese, Han Chinese		r2 = 0.93 with a missense variant (I254V)	(26, 33)
BD	rs61752717	*MEFV*	2.65	Turkish, Japanese		Missense variant (M694V), which increases response to LPS	(35)
BD	rs7203487	*IRF8*	1.39	Turkish, Iranian			(26)
BD	rs142105922	*IRF8*	1.61	Turkish, Japanese			(26)
BD	rs11117433	*IRF8*	1.59	Turkish			(26)
BD	rs681343	*FUT2*	1.30	Iranian, Turkish	Turkish	r^2^ = 1 with a nonsecretor allele (rs601338)	(34)
BD	rs913678	*CEBPB-PTPN1*	1.32	Turkish, Iranian	Han Chinese		(26, 33)
Sarcoidosis	rs3762318	*IL23R-C1orf141*	1.65	Japanese	Czech	Decreases expression	(36)
Sarcoidosis	rs12069782	*IL23R-C1orf141*	1.24	German			(37)
Sarcoidosis	rs6748088	*FAM117B*	1.18	German			(37)
Sarcoidosis	rs1499506	*MAGI1*	1.98	African American		Associated with ocular sarcoidosis	(38)
Sarcoidosis	rs223498	*NFKB1-MANBA*	1.19	German			(37)
Sarcoidosis	rs4921492	*IL12B*	1.20	German			(37)
Sarcoidosis	rs715299	*NOTCH4*	1.14	African American			(39)
Sarcoidosis	rs2302006	*CCL24*	1.31	Japanese	Czech	Decreases expression	(36)
Sarcoidosis	rs3779419	*STYXL1-SRRM3*	1.37	Japanese	Czech	Increases *POR* expression	(36)
Sarcoidosis	rs2789679	*ANXA11*	1.67	German	African American, European American, Han Chinese	High LD with a missense variant R230C	(39, 40)
Sarcoidosis	rs479777	*CCDC88B*	1.18	German			(37)
Sarcoidosis	rs653178	*SH2B3-ATXN2*	1.19	German			(37)
VKH disease	rs117633859	*IL23R*	1.82	Han Chinese	Singaporean, Japanese	Decreases expression in PBMCs	(41–43)
VKH disease	rs442309	*ADO-ZNF365-EGR2*	1.37	Han Chinese	Thai, Japanese		(41–43)
AAU	rs79755370	*IL23R*	1.80	European			(44)
AAU	rs2032890	*ERAP1*	1.51	European			(44)
BCR	rs7705093	*ERAP2*	2.20	Dutch, Spanish	British	rs10044354 T (r^2 =^ 0.98) increases ERAP2 in B-cell lines	(45)
BCR	rs2287987-rs10044354	*ERAP1, ERAP2^2^*	2.75	Dutch, Spanish		Decreases *ERAP1* expression	(46)
						Increases *ERAP2* expression	(45)
BCR	rs150571175	*TECPR2*	6.10	Dutch, Spanish			(45)
Crohn’s disease uveitis	rs4766697	*RBM19*	3.31	United States^3^			(47)

^1^Risk allele homozygotes of rs17482078 showed genome wide significance in BD patients with uveitis.

^2^Haplotype analysis of rs2287987 in the ERAP1 locus and rs10044354 in the ERAP2 locus showed disease susceptibility for BCR.

^3^Patients with IBD who were enrolled in the cedars-Sinai IBD Research Repository (MIRAD) were evaluated.

OR, odds ratio; Ref, reference,; BD, Behçet’s disease; VKH disease,: Vogt-Koyanagi-Harada disease; AAU, acute anterior uveitis; BCR, birdshot chorioretinopathy.

A GWAS in the Han Chinese population reported a susceptibility locus of *STAT4* and *TNFAIP3* (29, 32). Imputation of Turkish GWAS data additionally identified *CCR1*, *KLRC4*, and *ERAP1* (27). Meta-analysis of the *IL12A* susceptibility in a mixed population with Turkish GWAS data reached a genome-wide significant association level (31). In 2017, Takeuchi et al., conducted a large-scale genetic analysis, including 3,477 patients and 3,342 controls from Turkish, Japanese, and Iranian populations using the Immunochip (Illumina), which has 200,000 markers designed from previous GWASs of other immune-related diseases. This dense genotyping of immune-related loci identified six novel loci, *IL1A-IL1B*, *RIPK2*, *ADO-EGR2*, *LACC1*, *IRF8*, and *CEBPB-PTPN1* (26). Another Immunochip study in the European Spanish population identified a novel association in the locus of *JRKL-CNTN5* (24). The disease susceptibility of ancestry specific FUT2 non-secretor alleles, which influence gut microbiome composition and susceptibility to viral and bacterial infections, was reported in Turks, Japanese and Iranian (26, 34, 51–53). A targeted sequencing approach revealed genome-wide association of a missense variant of *MEFV* M694V, which is one of the causative mutations for familial Mediterranean fever (35).

### Sarcoidosis

Sarcoidosis is a systemic inflammatory disease characterized by non-caseating granuloma formation affecting multiple organs, such as lungs, eyes, skin, and heart (54). In a Danish and Finnish twin cohort study, when one proband of monozygotic twins has sarcoidosis, the risk of the second twin developing sarcoidosis is 80 times higher than in the general population. This observation indicates that genetic factors are involved in the development of sarcoidosis (55).

The MHC region also confers the strongest susceptibility to sarcoidosis. Among the HLA genes, multiple allele forms of *HLA-DRB1* (HLA-DRB1*03, 11, 12, 14, and *15*), which is an MHC class II molecule, and *BTNL2* have been associated with sarcoidosis (56–61). A GWAS in the German population identified genome-wide association in *ANXA11*, which has since been confirmed in Han Chinese, European American, and Portuguese populations (40, 59, 62–64). Meta-analysis of SNPs in the *ANXA11* locus, rs2573346 T and rs2789679 T showed protective association (OR;0.66 and 0.70, respectively) (65).

An African ancestry GWAS additionally identified susceptibilities in the loci of *CCDC88B*, and *NOTCH4*, and suggestive associations (1 × 10^−5^>P>5 × 10^−8^) in the loci of *RAB23*, *OS9*, and *XAF1* (66). In 2015, an Immunochip genetic study was performed in the European population, and four loci of *SH2B3-ATXN2*, *IL12B*, *NFKB1-MANBA*, and *FAM117B* were identified (37). Recently, a GWAS in a Japanese cohort with replication in independent samples from Japan and the Czech Republic newly identified *CCL24* and *STYXL1-SRRM3* and confirmed disease susceptibility of *IL23R* (36). GWAS of ocular sarcoidosis was reported by Garman et al., and the association of the locus of *MAGI1* in African Americans was identified, which also showed suggestive association when comparing ocular-sarcoidosis to non-ocular sarcoidosis (38).

### Vogt-Koyanagi-Harada Disease

Vogt-Koyanagi-Harada (VKH) disease is a systemic immune-mediated disorder that affects melanocytes contained in pigmented tissues, such as the uvea, inner ear, meninges, skin, and hair (67). Although the etiology of VKH disease is still unknown, it is thought that certain environmental factors, for instance, viral infection, provoke aberrant activation of the immune response through Th1 and Th17 pathways in individuals with a predisposing genetic background.

As in other non-infectious uveitis cases, the involvement of the MHC region has been reported. Disease susceptibility has been reported for MHC class II genes, such as *HLA-DR4*/*DRw53*, and *HLA-DQ4* In VKH disease (68). A systematic review of the association of HLA-DR4/HLA-DRB1*04 from 21 studies including 1853 patients with VKH disease and 4164 controls showed an odds ratio (OR) of 8.42 (95% CI, 5.69–12.45) with interethnic heterogeneity (69). The suballele analysis showed increased risk in HLA-DRB1*04:04 (OR=2.57), HLA-DRB1*04:05 (OR=10.31), and HLA-DRB1*04:10 (OR=6.52), but a protective effect in HLA-DRB1*04:01 (OR=0.21) (69).

A GWAS of a Chinese cohort including 1,538 cases and 5,603 controls reported disease susceptibility loci of *IL23R* and *ADO-EGR2* (41). Disease susceptibility of *IL23R* was confirmed in the Han Chinese Singaporean and Japanese populations, and *ADO-EGR2* was confirmed in the Japanese and Thai populations (42, 43).

### Acute Anterior Uveitis

Acute anterior uveitis (AAU) is a uniocular fibrinous iridocyclitis that develops acutely with fibrin precipitation and anterior chamber hypopyon. AAU is the most common cause of non-infectious uveitis in European countries, affecting 0.2% of the general population (70–73). Spondyloarthropathies (SpAs) comprising ankylosing spondylitis (AS), psoriatic arthritis, reactive arthritis, and inflammatory bowel disease are frequently found in AAU patients (74). Especially, AS is the most common condition with 30%–50% prevalence in patients with AAU (75).

AAU has been known to be associated with HLA-B*27 (76). HLA-B*27 is positive in > 50% of AAU cases in the Caucasian population compared to 8%–10% of the general population. Another study reported the frequency of HLA-B*27 positive was 81.8% and 92.0% in patients with ophthalmologist-diagnosed AAU and self-reported AAU, respectively (44). In contrast, the prevalence of AAU is lower in the Asian population, which has a commensurately low frequency of HLA-B*27.

A genetic analysis by Immunochip in the European population showed an OR of 66.8 for heterozygosity of HLA-B*27 and 130.6 for homozygosity, indicating that the HLA-B*27 molecule plays a key role in the pathogenesis of AAU (44). This study also identified susceptibility outside the MHC region, *IL23R*, *ERAP1*, 2p15 (44). A recent GWAS of AAU in the European population found genome-wide association at rs9378248 in *HLA-B*. The GWAS also found suggestive association in the previously reported locus of *ERAP1* and novel loci of *NOS2, MERTK*, *KIFAP3*, *CLCN7*, *ACAA2*, and 5 intergenic loci (77).

### Birdshot Chorioretinopathy

Birdshot chorioretinopathy (BCR) is a bilateral chronic posterior uveitis with creamy ovoid choroidal spots called “birdshot” on the fundus (78). It is predominant in middle age and slightly more common in women by sex, and most patients are Caucasian, especially of northern European ancestry. Conversely, there are few studies from other races.

A strong association between BCR and HLA-A*29 has been reported. Of the HLA-A*29 subtypes, HLA-A*29:01 and HLA-A*29:02 are common in the general population (0.2% and 4.3% in the Caucasian population, respectively). HLA-A*29:02 is positive in > 95% of BCRs, indicating that this HLA allele is strongly involved in the development of BCRs. However, patients with HLA-A*29:01-positive BCR are rarely observed. Unlike other non-infectious uveitis involving HLA genetic predisposition, BCR does not show predominant extraocular manifestations (79).

In GWASs conducted in Dutch and Spanish populations, HLA imputation analysis identified the strongest susceptibility of HLA-A*29:02 with OR of 157.5 among all markers including HLA alleles, amino acid changes, and SNPs (45). From outside the MHC region, a genome-wide association was observed in *TECPR2* (45). In addition, a meta-analysis in the British population revealed susceptibility of *ERAP2* to BCR (45). A haplotype analysis revealed genome-wide significance of an *ERAP1-ERAP2* haplotype (45).

### Tubulointerstitial Nephritis and Uveitis Syndrome

Tubulointerstitial nephritis and uveitis syndrome (TINU) is a bilateral sudden-onset anterior uveitis accompanied by tubulointerstitial nephritis (80). An HLA analysis of acute tubulointerstitial nephritis (ATIN) including patients with TINU and drug hypersensitivity-related ATIN (D-ATIN) in the Chinese population identified genome-wide significant association of HLA-DQA1*01:04, and HLA-DRB1*14:05 in both TINU and D-ATIN and HLA-DQB1*05:03 in D-ATIN (81).

### Other Systemic Immune-Related Diseases

Uveitis is also observed in systemic immune-related diseases such as juvenile idiopathic arthritis (JIA) and inflammatory bowel disease (IBD). Several susceptibility genes have been identified in these diseases. However, the frequency of uveitis in these diseases is not high, i.e., 10–30% in JIA (82) and 2–4% in IBD (47). Therefore, whether the reported susceptibility genes are really involved in the pathogenesis of uveitis remains unclear.

Haasnoot et al. compared patients with JIA with and without uveitis and reported that amino acid serine at position 11 (serine 11) of HLA-DRβ1 was strongly correlated with uveitis in JIA (OR=2.60, P=5.43×10^-10^) (82). In addition, the susceptibility of serine 11 was found only in girls and not in boys (P_girls_=7.61×10-10, P_boys_=0.18). Epidemiologically, uveitis is more prevalent in girls with JIA (83), and this genotype-sex interaction may be a factor to elucidate this sexual dimorphism.

Taleban et al. identified the susceptibility of a region of *RBM19* according to GWAS of uveitis in IBD (47). While the novel susceptibility locus was detected, none were identified from the several known regions of IBD in the study, due in part to the small sample size and low statistical power. Although little is known on the function of RBM19, it is probably involved in the regulation of ribosomal biogenesis. Replication studies are warranted to confirm the susceptibility of *RBM19* to IBD uveitis.

## Immunogenetics of Non-Infectious Uveitis

### MHC

**Figure 1:**
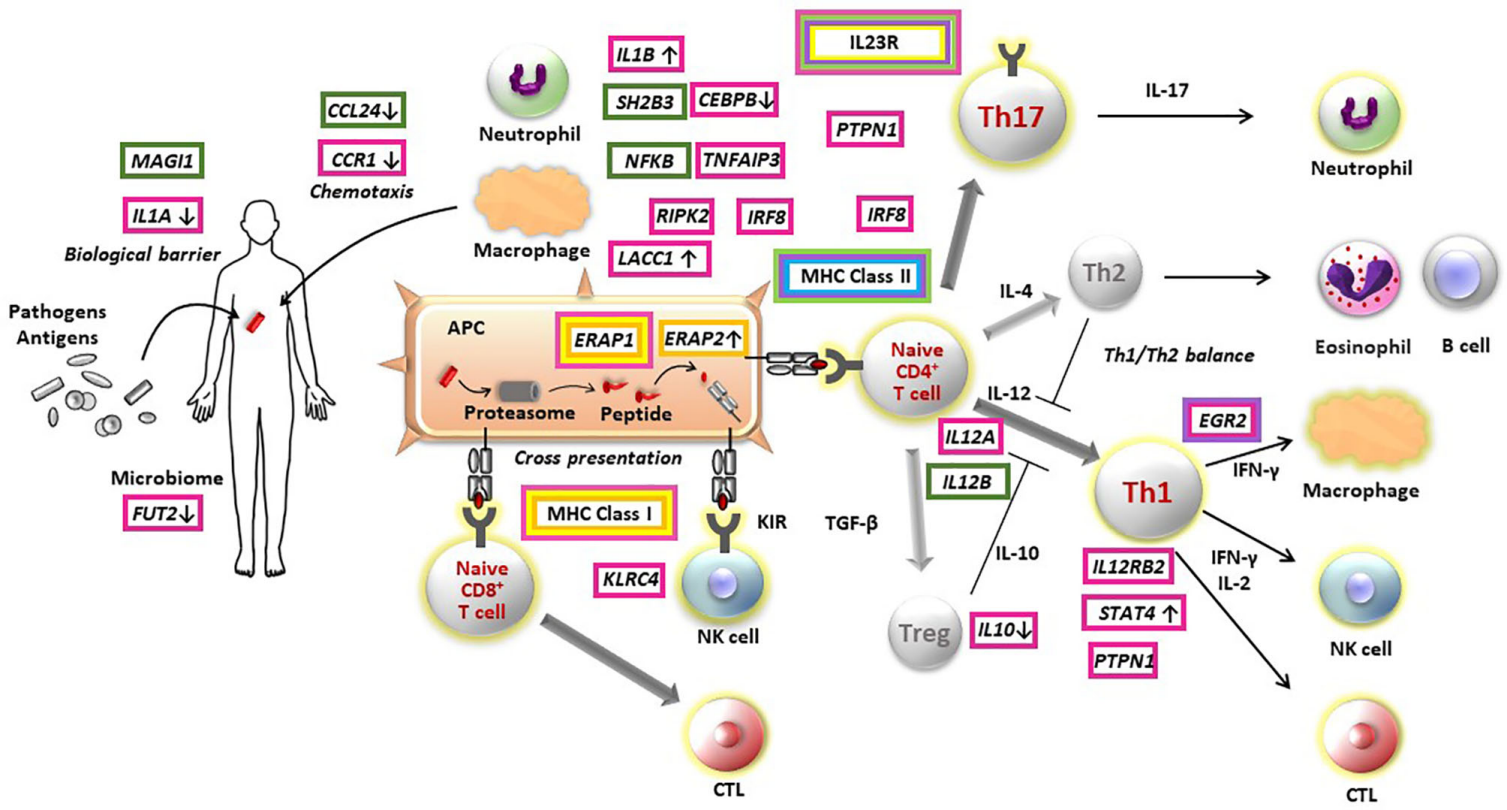
Pathogenesis of non-infectious non-infectious uveitis elucidated by immunogenetic findings. Susceptibility genes of Behçet’s disease (red boxes), sarcoidosis (green boxes), Vogt-Koyanagi-Harada disease (purple boxes), acute anterior uveitis (yellow boxes), birdshot chorioretinopathy (orange boxes), and tubulointerstitial nephritis and uveitis syndrome (light blue boxes) are indicated in the figure. The direction on gene expression or function of risk alleles are described with arrows, where identified. APC, antigen presenting cell; CTL, cytotoxic T cell; NK, natural killer cell; Treg, regulatory T cell.

The genetic findings from several GWASs provide evidence to elucidate pathogenesis of non-infectious uveitis (**Figure 1**). To date, studies have consistently found that all major non-infectious uveitis diseases were genetically most strongly associated with HLA alleles, classified into the MHC class I and MHC class II. MHC molecules play an essential role in the adaptive immune system by presenting peptide antigens derived from pathogens and self-proteins on the surface of cells, specialized antigen-presenting cells for MHC class II and nearly all cells for MHC class I.

From the point of view of MHC susceptibility, non-infectious uveitis can be divided into two groups. BD, AAU and BCR are associated with MHC I molecules and sarcoidosis, VKH and TINU are associated with MHC II molecules.

The MHC class I molecule is involved in cell-mediated immunity by presenting antigens to CD8-positive cytotoxic T cells. In contrast, MHC class II molecules influence humoral immunity by presenting antigens to CD4-positive helper T cells. However, there is not a one-to-one correspondence between each non-infectious uveitis and abnormality in one immune pathway. For instance, for BD, although the strong MHC class I association suggests a role of CD8-positve T cell-mediated immunity, the associations of *IL23R-IL12RB2* and *IL10*, identified by the initial GWAS for BD, are genes involved in the immune pathway of CD4-positive T cells (22, 23). In addition, several genes identified thereafter were genes involved in innate immunity (26, 27). These findings indicate that abnormalities in multiple immune pathways intricately contribute to each disease pathogenesis.

In BD, independent risk or protective variants of HLA-B amino acids identified by the stepwise conditional analysis were mainly located within the antigen-binding grove of the HLA molecule (21, 84).. The strongest association was observed for threonine at position 97, including HLA-B*51, followed by leucine at 116, glutamic acid at 152, and phenylalanine at 67, with independent associations (21). Amino acid position 97 also showed the most significant association in patients with AS and AAU compared with patients with AS without AAU (77). After the conditioning on amino acid position 97, independent associations of positions 67 and 70 in HLA-B were revealed (77). Amino acid positions 116, 67, and 97 located in the HLA-B antigen-binding groove are known to be key determinants that remotely influence the HLA-B alleles binding affinity of KIR3DL1 and KIR3DS1 receptors (85). These findings further implicate peptide binding on MHC molecules in the pathogenesis of non-infectious uveitis.

While MHC class I molecules are present in all nucleated cells and present with intracellular antigens, MHC class II molecules are expressed on dendritic cells, macrophages, and B cells and present extracellular antigens such as bacteria, viruses, and soluble proteins to CD4-positive T cells. Considering that flu-like symptoms are often observed in the prodromal phase of patients with VKH, a viral infection is considered to be a trigger for disease development. In this hypothesis, MHC class II molecules that recognize specific viral antigens that may elicit an immune response by cross-reaction with self-proteins associated with melanocytes (67). Patients with VKH are sensitized to melanocyte antigens, and the melanocyte peptide binding capacity of the *HLA-DRB1* allele is thought to influence disease susceptibility. Susceptible *HLA-DRB1* alleles such as HLA-DRB1*0405 may broadly recognize melanocyte associated antigens (86), whereas protective alleles such as HLA-DRB1*0401 showed a vulnerable binding capacity (87).

### Genetic Interaction With MHC Class I

#### ERAP1/ERAP2

*ERAP1*, which is a common susceptibility gene to AAU and BD, and *ERAP2*, identified in the GWAS of birdshot retinopathy, encode endoplasmic reticulum aminopeptidase 1 and 2 proteins (ERAP1 and 2), respectively. ERAP1 and ERAP2 are enzymes that trim proteasome-processed precursor peptides in the ER for loading onto the MHC I molecule. The rs1044354 C allele in high linkage disequilibrium (LD) with the rs7705093 T allele, which is associated with susceptibility to BCR, is associated with high mRNA expression of *ERAP2* in LCL cells and homozygotes of the non-risk allele showed little or no protein expression in B cells from controls (45).

A missense mutation in *ERAP1* p.Asp725Gln showed susceptibility in patients with BD uveitis only under HLA-B*51 positivity, and *ERAP1* susceptibility was abolished in HLA-B*51-negative patients (27). Analysis of *ERAP1* haplotypes in BD showed strong susceptibility to Hap10 in HLA-B*51-positive patients with OR of 10.96 compared with those with neither HLA-B*51 nor *ERAP1* Hap10 (88). It is suggested that polymorphisms in *ERAP1* Hap10 affect trimming efficiency and peptide length (89).

Epistasis between *ERAP1* and MHC class I was also observed in other MHC-I-opathies, such as AS and psoriasis (74, 90, 91). In contrast to BD, *ERAP1* p.Asp725Gln acts protectively for AS under HLA-B*27 positivity and also for psoriasis under HLA-Cw6 positivity (27). The differential effects of *ERAP1* variants in conjunction with disease-specific MHC class I alleles among these diseases may be explained by differential availability of disease-specific peptides.

In BD, an inefficient ERAP1 enzyme could fail to degrade a disease-promoting peptide. Majority of the position 2 of the peptidome bound to HLA-B51:01 is Ala with lower affinity or Pro with higher affinity for HLA-B*51:01 (92). Guasp et al. compared the peptidome of HLA-B*51 trimmed by homozygotes of ERAP1 Hap10 and ERAP1 Hap1/Hap8 and found that Hap10 increased the Ala/Pro ratio at position 2 in addition to affecting the amino acid length distribution (92). In HLA-B51/ERAP1 Hap10 individuals, the low affinity of the HLA-B51-peptide complex is assumed to enhance the activation of NK cells.

On the other hand, in AS and psoriasis, an inefficient ERAP1 enzyme could fail to produce a disease-promoting peptide. A gene interaction was analyzed between a nonsynonymous SNP located in *ERAP1* rs30187, which showed suggestive association with AAU, and HLA-B*27. Genome-wide association was revealed when comparing patients with HLA-B*27-positive AS with AAU and without AAU. In contrast, *ERAP1* susceptibility disappeared when comparing patients with HLA-B*27-negative AS with AAU and without AAU (77).

Both a tag SNP of *ERAP1* Hap10 haplotype, which is associated with decreased ERAP1 protein expression and enzymatic activity, and rs10044354, which is associated with increased ERAP2 expression, showed strong association in patients with HLA-A*29-associated BCR. The combined haplotype of *ERAP1-ERAP2* resulted in a genome-wide association with BCR by meta-analysis (OR 2.75, P=2.6 × 10^-8^) (46).

#### Killer Immunoglobulin-Like Receptors

Killer cell immunoglobulin-like receptors (KIR) are receptors on natural killer cells, which ligate to the MHC class I molecules (93). The KIR family clustered on chromosome 19q13.4 is divided into activating KIRs (aKIRs) and inhibitory KIRs (iKIRs) according to the length of the cytoplasmic tail, short (S) and long (L), respectively.

Although the role of KIRs may be critical in the pathogenesis of BD, an MHC-I-opathy, GWASs of non-infectious uveitis have not revealed genome-wide association with KIRs, and many candidate gene approach studies did not find a statistical association with KIRs (94–96). A recent study showed the frequency of KIR3DL1*004, which encodes a misfolded protein of this iKIR, was decreased in patients with BD with or without HLA-B*51 (97). Evaluating functional *KIRD3L1/S1* alleles, increasing risk of BD was observed in individuals with *KIR3DL1^LOW^/KIR3DS1*genotypes (OR = 2.47), and individuals with *KIR3DL1^HIGH^/KIR3DL1^NULL^* genotypes showed a protective effect to the disease (OR=0.53) (98).

Evaluated by absence or presence of 16 KIR genes, KIR3DL1^-^/2DS3^-^ was significantly associated with increased risk of uveitis in patients with AS (96).

The association between KIR and VKH disease has also been evaluated despite the lack of evidence of association with MHC class I. No statistical difference of KIRs was identified in Mestizo individuals living in Southern California by Levinson et al. (99). They also conducted KIR analysis in the Japanese population, a more genetically homogeneous population. A high frequency of aKIRs, KIR3DS1, 2DS1, and 2DS5 and a low frequency of an iKIR of KIR3DL1 were observed in Japanese patients with VKH disease compared with healthy controls (42.2% vs 21.4% and 76.9% vs 98.8%, respectively) (100). Sheereen et al. reported a high frequency of KIR2DS3 and KIR Bx genotypes, which includes 2DS2, 2DS3, and 2DS4, in Saudi Arabian patients with VKH disease (101). They also found weak associations of MHHHC class I genes, HLA-Cw*14 and -Cw*17, and protective association in the combination of KIR2DL2/2DL3 and HLA-C1 (101).

Immunogenetics of the MHC genes, ERAP1/ERAP2, and KIRs strongly suggest that pathways influencing peptide-MHC class I binding affinity and regulation of CTL and NK cell activation play crucial roles in the pathogenesis of several causes of non-infectious uveitis.

### IL-23/Th17 Pathway

The most common susceptibility gene outside the MHC region is IL23R, encoding interleukin 23 receptor (IL-23R), identified from GWASs of several major non-infectious uveitis causes, such as BD, sarcoidosis, VKH, and AAU. Although disease risk alleles of BD and sarcoidosis were located in the IL23R locus, those are not in the same LD block, and the BD-associated SNP was not associated with susceptibility to sarcoidosis (36).

The IL-23 receptor is expressed on the surface of Th17 cells and involved in the maintenance of Th17 homeostasis and IL-17 production with recognition of IL-23 as a ligand (102). IL-23 is a key checkpoint for naïve T cell to differentiate into pathogenic Th17 cells and also contribute to homeostasis of Th17 cells and Th17 cell cytokine production, such as IL-17, IL-6, interferon γ, and granulocyte macrophage colony-stimulating factor (103).

In vivo studies on each non-infectious uveitis have supported these genetic findings. The serum IL-17 level was increased in patients with BD uveitis, and the IL-23 level was also elevated in peripheral blood mononuclear cells (PBMCs) from patients with active BD and VKH disease compared with inactive patients and healthy controls (104). The proportions of IL-17A+ cells contained in circulating memory CD4+ T cell populations were increased in patients with sarcoidosis (105). Increased IL-17A+ cells were also observed in the lamina propria from biopsies containing granulomas compared with those from biopsies from healthy controls or non-granulomas biopsies (105). Elevated IL-23p19 mRNA and IL-23 protein production levels in PBMCs, increased serum IL-23 level, and increased IL-17 level in polyclonally stimulated PBMCs and CD4+ T cells were reported in patients with VKH disease (106). Serum IL-23 level was elevated in patients with BCR with active disease naïve to systemic treatment compared with that in controls (107). These data suggest that IL-23/Th17 pathway cells, in which the IL-23 receptor is involved, are the basis of a common pathophysiology of non-infectious uveitis, regardless of inflammatory properties, such as serous and granulomatous properties, or the class of MHC association with the disease.

### Biological Defense

Functional studies have revealed how susceptible polymorphisms affect the expression or function of genes in the locus. The risk allele of rs1518111 for BD, the lead SNP in the IL10 locus, is associated with decreased IL10 expression in monocytes (23). This polymorphism may decrease IL-10 production, which is an anti-inflammatory cytokine, leading to Th1 polarization. The risk allele of rs7574070 is associated with increased gene expression of *STAT4*, which plays a role in the differentiation of naïve T cells into Th1 and Th17 cells (108). These functional findings of risk alleles may lead to hyperinflammation of immune pathways, contributing to the development of non-infectious uveitis.

Interestingly, it is reported that some risk alleles for BD in gene loci involved in innate immunity act in the opposite direction, which could suppress inflammation and impair the biological defenses against pathogens. For example, risk alleles of SNPs in the *CCR1* locus reduce CCR1 expression levels and monocyte migration in PBMC from healthy controls (48). The risk allele of the lead SNP, rs4402765 in the locus of *IL1A-IL1B*, is associated with reduced production of IL-1α, which is an important contributor to biological defense in the skin, but interestingly, it is also associated with increased production of IL-1β (26), an important antimicrobial inflammatory molecule. The evidence that a BD-associated genetic locus downregulates IL-1α production in conjunction with upregulation of IL-1β raises the intriguing possibility that the combination of impaired barrier function and excessive inflammatory response to invading microorganisms contribute to BD risk.

The risk allele of rs117633859, a disease-associated SNP for sarcoidosis in both the Chinese and Japanese populations, and VKH disease in the Han Chinese population reduced IL23R transcription activity in HEK-293A cells and *IL23* expression (36, 41). This suggests that impairing host defense against pathogens by downregulating the IL-23R/Th17 axis plays an important role in the development of VKH disease and sarcoidosis.

Although consensus has yet to be reached, it has been thought that transient or occult microbial infection may play a role as a trigger in disease onset of non-infectious uveitis (67, 109). These immunogenetic findings may help clarify the involvement of pathogens in the development of non-infectious uveitis.

## Bench to Bedside

### Genetics and Clinical Manifestation

Disease susceptibility genes identified by genetic approaches, such as GWAS, help clarify pathways leading to the onset of non-infectious uveitis. Moreover, the identified genes and pathways have the potential to be applied to drug discovery as targets for new treatments and for prediction of prognosis and drug efficacy based on the genetic attributes of individual patients. Because subgrouping by clinical manifestations results in analyses with smaller sample size that may limit the detection of statistical associations, the evidence connecting genetics to clinical manifestations has been seldom reported.

Although the evidence connecting genetics to clinical manifestations has not been sufficiently reported yet, some studies showed that HLA alleles may affect disease clinical features and prognosis. Uveitis is more common in patients with HLA-B*51-positive BD compared with those with HLA-B*51-negative BD (110). In addition, HLA-A*26-positive patients have a poor visual prognosis (111).

In patients with sarcoidosis, HLA-DRB1*04 is associated with eye involvement (112). The association between HLA alleles and extraocular manifestations of non-infectious uveitis has been reported. HLA-DQB1*06:01 was more common in Japanese patients with cardiac sarcoidosis (113). HLA-DRB1*04/*15 increased the risk of extrapulmonary involvement (including uveitis) in the European population (114). HLA-DRB1*03:01 showed association with Löfgren syndrome, a subtype of sarcoidosis with acute onset of systemic inflammation disorder (58). Mackensen et al. performed HLA analysis on European patients with TINU and TIN and reported that the incidence of HLA-DRB1*01:02 was increased only in patients with TINU compared with those with TIN and healthy controls (115).

Meguro reported that, in Japanese and Czech populations, the OR of the rs4728493 risk allele at the *CCL24* locus decreases with the progression of the sarcoidosis chest X-ray (CXR) when compared in the CXR stage 0 + I, stage II, and stage III + IV subgroups (36). The highest OR was observed for susceptible SNPs in both *CCL24* and *STYXL1-SRRM3* in the acute systemic Lofgren’s syndrome compared with sarcoidosis with any chest X-ray stage in the Czech population (36). The risk allele of rs4728493 was associated with decreased *CCL24* expression in fibroblasts and skin. CCL24 is an eotaxin, which is upregulated by Th2-biased immune responses; thus, these findings indicate that Th1 polarization associated with decreased expression of CCL24 might be important in onset and maintenance of the early stage and acute subtype of sarcoidosis (36, 116).

### Treatment Based on Immunogenetics

Anti-inflammatory treatment by topical or systemic administration of corticosteroids and immunosuppressants have been the basic treatment for non-infectious uveitis (117). In recent years, the development of biologics targeting specific molecules involved in pathological conditions has progressed, and multiple biologics have been used in the treatment of non-infectious uveitis (118). In particular, anti-TNF reagents have significantly improved the visual acuity prognosis of patients with intractable uveitis due to their strong anti-inflammatory effect. Multicenter double-masked randomized clinical trials of adalimumab for non-infectious uveitis with insufficient control by corticosteroids (VISUAL I, VISUAL II, and VISUAL III) reported efficacy of adalimumab with a lower risk of ocular flare and visual impairment (119–121).

However, while the efficacy of TNF inhibitors is noteworthy, there are still cases of refractory non-infectious uveitis who do not respond to TNF inhibitors. Therefore, it is desirable to establish treatment based on the pathogenesis of each disease and the genetic factors of the individual as precision medicine.

IL-1β encoded from IL1B, identified in the BD Immunochip study, is an inflammatory cytokine and plays an important role in biological defense through activating NF-κB. In healthy donor PBMCs homozygous for rs4402765, the lead SNP for BD in the IL1B region, IL-1β was expressed higher than in cells homozygous for the non-risk allele (26). IL-1 inhibitors, anakinra and canakinumab, showed a significant reduction of ocular attacks, resolution of active retinal vasculitis, and a decrease in steroid dosages in patients with BD uveitis (122). Other studies with a small sample size also reported the effectiveness of IL-1 inhibitors in BD uveitis (123–125).

Other candidate treatment targets for non-infectious uveitis may be molecules involved in the IL-23R/Th17 pathway according to the susceptibility of IL-23R in BD, sarcoidosis, VKH disease, and AAU as previously described. Ustekinumab, an inhibitor of the p40 subunit of IL-23, effectively suppressed eye inflammation in patients with BD in concurrence with its efficacy in psoriasis (126–128). Ustekinumab also showed effectiveness for oral ulcers in patients with BD, which were resistant to colchicine (129, 130). IL-6 is a pleiotropic cytokine involved in the differentiation of TH17 cells and activation of STAT3 in the JAK-STAT pathway (131). A multicenter study including 11 patients with BD treated by tocilizumab, an IL-6 inhibitor, reported 8 of 11 achieved a complete remission at 9.5 months with two withdrawn due to severe infusion reaction (132). Secukinumab is a fully human monoclonal antibody that binds to IL-17A, leading to neutralization. Hueber et al. reported that secukinumab suppressed ocular inflammation in 11 of 16 patients with active non-infectious uveitis (133). Letko et al. reported efficacy and safety for the treatment of intravenous secukinumab in remission rates of non-infectious uveitis compared with subcutaneous administration (134, 135). However, three multicenter RCTs, including 118 patients with BD uveitis, 31 patients with active non-BD uveitis, and 125 inactive non-BD uveitis reported that no statistical effectiveness in recurrence of non-infectious uveitis was observed in all three studies compared with placebo groups (136). The authors noted that patients in both groups were treated with high doses of concomitant immunosuppressive drugs with and without secukinumab in the study and that these may have contributed to the lack of significant differences between secukinumab and placebo groups (136). In addition, for non-infectious uveitis with variable clinical manifestation, a larger sample size and longer observation period may be necessary to accurately evaluate the anti-inflammatory effect of secukinumab.

## Conclusions

We have described the immunogenetics of major non-infectious uveitis, focusing on the genetic findings by GWASs. Similar to other immune-related disorders, HLA genes are the strongest genetic factors in major non-infectious uveitis, and evidence from studies on ERAP1/ERAP2 and KIR suggests that the binding of peptides to MHC molecules and immune response through MHC antigen presentation are considered central to these pathological conditions. Outside of the MHC, the fact that disease susceptibility of IL23R has been reported for several non-infectious uveitis conditions at a genome-wide significant level, suggests that the IL-23/TH17 pathway may be a common basis for the pathogenesis of non-infectious uveitis. Based on the immunogenetics of non-infectious uveitis, it is expected that the development of molecular-targeted drugs based on the pathophysiology of non-infectious uveitis and selection of treatment options based on the genetic attributes of individual patients are required to increase efficacy and reduce side effects.

## Author Contributions

MT wrote this manuscript. NM and SO revised manuscript. All authors contributed to the article and approved the submitted version.

## Funding

This study was supported by grants from Ministry of Health, Labour and Welfare, Japan (H29-Nanchi-ippan-050).

## Conflict of Interest

The authors declare that the research was conducted in the absence of any commercial or financial relationships that could be construed as a potential conflict of interest.
